# Estradiol Valerate Pretreatment in Short Protocol GnRH-Agonist Cycles versus Combined Pretreatment with Oral Contraceptive Pills in Long Protocol GnRH-Agonist Cycles: A Randomised Controlled Trial

**DOI:** 10.1155/2015/628056

**Published:** 2015-04-02

**Authors:** Krzysztof Lukaszuk, Joanna Liss, Michal Kunicki, Waldemar Kuczynski, Ewa Pastuszek, Grzegorz Jakiel, Lukasz Plociennik, Krzysztof Zielinski, Judyta Zabielska

**Affiliations:** ^1^INVICTA Fertility and Reproductive Centre, 80-172 Gdansk, Poland; ^2^Department of Nursing, Medical University, 80-952 Gdansk, Poland; ^3^INVICTA Fertility and Reproductive Centre, 00-019 Warsaw, Poland; ^4^Centre for Reproductive Medicine KRIOBANK, 15-879 Białystok, Poland; ^5^Department of Gynecology and Oncological Gynecology, Medical University, 15-276 Białystok, Poland; ^6^Department of Obstetrics and Gynaecology, Medical Centre of Postgraduate Education, 01-813 Warsaw, Poland

## Abstract

The strategy of in vitro fertilization (IVF) procedures relies on the increasing pregnancy rate and decreasing the risk of premature ovulation and ovarian hyperstimulation syndrome. They are also designed to avoid weekend oocyte retrievals. Combined oral contraceptive (OC) pills are among the medicines used to accomplish these objectives. Alternatively, estradiol can be used instead of OC to obtain similar results. The aim of our study was to compare the differences in pregnancy rates (PRs), implantation rates, and miscarriage rates between a short agonist protocol with estradiol priming and a long protocol with combined OC. Of the 298 women who participated in this study, 134 achieved clinical pregnancies (45.0%). A higher PR (58.4%, *n* = 80, compared to 40.3%, *n* = 54) was achieved in the long protocol after OC pretreatment group. The implantation rate was also higher for this group (37.8% versus 28.0%; *P* = 0.03). The miscarriage rate was 15.0% (*n* = 12) for the long protocol after OC pretreatment group and 20.4% (*n* = 11) for the short agonist group (*P* = 0.81). The short agonist protocol required a 5.7% lower human menopausal gonadotropin (hMG) dosage than the long protocol but surprisingly the number of oocytes retrieved was also smaller.

## 1. Introduction

IVF cycles are sequences of medical procedures that increase effectiveness of IVF procedure and decrease the risk of premature ovulation and ovarian hyperstimulation syndrome. They are also designed to avoid weekend oocyte retrievals. Combined oral contraceptive (OC) pills are among the medicines used to achieve these objectives. Alternatively, estradiol can be used instead of OC to obtain similar results. Using estradiol, Huirne reported similar findings in addition to a reduction in large follicle occurrences prior to day eight [1]. In contrast, it has been shown that oestrogen administration (at a dosage of 4 mg/day) does not suppress LH or FSH serum levels [2].

The use of OC for pituitary suppression in GnRH antagonist cycles has been associated with slower follicular growth and lower serum estradiol levels during the early portion of the cycle. These changes result in a longer duration of rFSH stimulation and a greater total rFSH consumption than observed in antagonist cycles without pretreatment [2].

The use of OC in agonist cycles is especially useful for decreasing the risk of cyst formation [3]. This factor is important because of the poor stimulation outcome and the reduced pregnancy rate (PR) that occur in a cycle when an ovarian cyst is present [4]. Estradiol may also prevent the weekend oocyte retrievals. The advantages of ovarian stimulation with a GnRH-agonist instead of GnRH-antagonist cotreatment were observed in our clinics. For example, antagonists generate weaker pituitary suppression, which results in sporadic premature ovulation and thus we decided to focus on agonist protocols.

The aim of our study was to compare the differences in the PR, implantation rate, and number of miscarriages between a short agonist protocol with estradiol priming and a long protocol with combined OC.

## 2. Materials and Methods

Participants were recruited from the Invicta Clinics in Gdansk, Warsaw, and Slupsk between July 2013 and January 2014.

The inclusion criteria were as follows: women younger than 40 years old, an AMH level greater than 0.6 ng/mL, a body mass index between 18 and 29 kg/m^2^, and undergoing a first or second treatment cycle of IVF with intracytoplasmic sperm injection (ICSI).

The exclusion criteria were endometriosis and preimplantation diagnosis cycles.

After providing written consent, the participants were divided into one of the two study groups using our block randomization software to ensure complete allocation concealment. The doctors performing the oocyte retrieval procedure and the embryologists involved were blind to the treatment groups.

The women had their serum AMH, DHEA, SHBG, testosterone, LH, FSH, and E2 levels measured between days 2 and 5 of their cycle and within three months prior to the intended IVF treatment.

In the long GnRH agonist regimen, patients were pretreated with a combination of OC (Ovulastan, Adamed, Pabianice, Poland) from the 2nd to 4th day of the cycle. Beginning from the 14th day of the cycle, the pituitary was suppressed by administering 0.1 mg triptorelin (Ferring, Saint-Prex, Switzerland) every 2 days; this treatment continued for 2 weeks. Ovarian stimulation commenced with gonadotropin injections at a dosage of 150–225 IU/day starting from 2–4 day of cycle and continued with a daily dose of 0.1 mg triptorelin until the hCG injection (Choragon 5000 IU, Ferring GmBh, Kiel, Germany) was administered 36 hours before retrieval.

The criterion for ovulation trigger was based on the amount of estradiol per follicle (greater than 14 mm) being greater than 200 pg/follicle and the diameter of the largest follicle being greater than 20 mm.

For the short GnRH agonist regimen, patients were pretreated with 2 mg oral estradiol twice daily from the 20th day of the natural cycle until the 1st–4th day of the new cycle. Ovarian stimulation commenced with human menopausal gonadotropin (hMG) (Menopur, Ferring, Saint-Prex, Switzerland) injections at a dosage of 150–225 IU/day starting from 2–4 day of cycle, 2 days after discontinuation of estradiol administration, and continued with a daily dose of triptorelin 0.1 mg (Ferring, Saint-Prex, Switzerland) until the hCG injection (Choragon 5000 IU, Ferring, Saint-Prex, Switzerland) was administered 36 hours before retrieval. The hMG dose used depended on the AMH level and the woman's age.

Effective suppression was confirmed by assessing levels of E2, LH, and progesterone hormones and antral follicle count (AFC) on the first day of ovarian stimulation. Concentrations of LH < 4 mIU/mL served as confirmation of pituitary downregulation for both groups. Transvaginal ultrasound-guided oocyte retrieval (TVOR) was performed 36 hours after the hCG injection. Embryo transfer was performed under transabdominal ultrasound guidance 5 days after oocyte retrieval. In each case, two embryos, provided that two were available, were placed in the uterine cavity. A total of 300 mg of micronized progesterone (Lutinus, Ferring GmbH, Kiel, Germnay) and 6 mg of estradiol (Estrofem, Novo Nordisk, Hillerød, Denmark), divided into three doses, were administered vaginally every day starting from the day of oocyte retrieval. This treatment regime continued until there was a negative pregnancy test result or until the 8th week of gestation. The hCG level was measured 11-12 days after blastocyst transfer. Clinical pregnancy (detection of a fetal heartbeat on an ultrasound scan) was confirmed at 6 weeks and 1–3 days after embryo transfer. Embryo implantation was confirmed by a transvaginal ultrasound at 5 weeks and 3 days +/−2 days after embryo transfer. Miscarriage was confirmed by ultrasound up to 12 weeks after embryo transfer.

Prenatal tests according to the Fetal Maternal Foundation (FMF) were performed between the 11th and 14th weeks of pregnancy.

The primary outcome measurement was the clinical PR. The secondary outcome measurements were the implantation rate and the miscarriage rate. We also analysed the number of retrieved mature oocytes, total dose of gonadotropin used for ovarian stimulation, and length of stimulation.

### 2.1. Specimen Collection and Preparation

Fasting venous blood samples (7 mL) were collected aseptically without any additives between 8:00 AM and 12:00 PM on day 12 after embryo transfer. The blood was allowed to clot at room temperature, and the serum was separated by centrifugation. The samples were stored at −20°C until analysis.

### 2.2. Hormone Level Analysis

FSH (3.5–12.5 mIU/mL), LH (2.4–12.6 mIU/mL), estradiol (12.5–166 pg/mL), DHEA-S (98.8–340 *μ*g/dL), testosterone (0.29–1.67 nmol/L), and SHBG (26.1–110.0 nmol/L) levels were determined using electrochemiluminescence immunoassays (Cobas 6000, Roche). Because of the inconsistent results obtained using the Beckman Coulter AMH assays, the AMH (>1.40 ng/mL) level was determined using ELISA on a first-generation assay of AnshLab. The inhibin B level (>20 pg mL) was determined using the Beckman Coulter ELISA. The sensitivities of each of these assays were as follows: AMH (0.02 ng/mL), inhibin B (7.2 pg/mL), estradiol (5.00 pg/mL), FSH (0.100 mIU/mL), LH (0.100 mIU/mL), DHEA-S (0.100 *μ*g/dL), testosterone (0.087 nmol/L), and SHBG (0.800 nmol/L). The intra-assay and interassay coefficients of variation were less than 10% for all assays.

### 2.3. Power Calculation and Statistical Analysis

The STATISTICA data analysis software system (StatSoft, Inc. [2011]) version 10 (http://www.statsoft.com/) was used for the data analysis. The clinical characteristics of the groups were compared using the Mann-Whitney *U* test.

According to power calculations, a minimum of 160 cycles in each arm of the study is needed to show a significant difference in the PR, which was the primary outcome measurement of this randomized study. This minimum was calculated for a difference of 15% in the PR, which we obtained in pilot studies (data not published).

The significance level (#) was set at 0.05 with a power of 0.9. An interim analysis of the number of pregnancies achieved showed that there was a sufficient number of pregnancies to obtain the required power level. Hence, recruitment was terminated, and an analysis of the results was conducted. Pearson's Chi-square test and Fisher's test were used to assess differences in the rates of development between the groups. A value of *P* < 0.05 was considered statistically significant.

This study was approved by the Bioethics Committee in Gdansk.

## 3. Results

**Figure 1:**
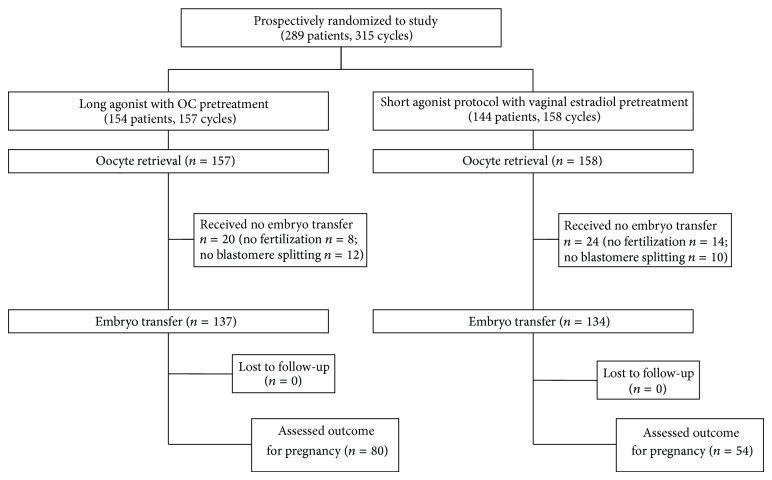
Flow chart diagram comparing the short agonist protocol with estradiol priming with the long protocol with combined OC.

During the study period, 298 cycles were included and randomized. Group 1 (long agonist cycle with OC) included 154 cycles, and group 2 (short agonist cycle with estradiol) included 144 cycles (Figure 1).

Table 1 presents the characteristics of the groups. No differences were found in the mean age, cause of infertility, duration of infertility, BMI, or hormone levels.

Table 2 presents the characteristics of the ICSI cycles. No differences were found in the mean number of oocytes retrieved, oocytes fertilized, or embryos transferred. Of the 298 women who were evaluated, 134 achieved clinical pregnancies (45.0%). A higher PR (58.4%) was achieved in Group 1 (Table 2). The implantation rate was also higher for Group 1 (37.8%; 28.0%; *P* = 0.03). The miscarriage rate was 15.0% for Group 1 and 20.4% for Group 2 (*P* = 0.81). We found that the short agonist protocol required a 5.7% lower hMG dosage than the long protocol, but surprisingly the number of oocytes retrieved was also smaller.

## 4. Discussion

The role of pretreatment in IVF stimulation has been widely discussed in the literature, and such pretreatment has been used for many years [2]. These pretreatments suppress the woman's own hormone production, and different types of steroids such as combined OC pills and oestrogens or progestogens alone are used. These hormones are used to minimize cyst formation and decrease pregnancy loss after implantation. OCs and other pretreatments are also very effective at ensuring that the retrieval of oocytes will occur during the work week.

The main decisions that must be made during the clinical portion of IVF can be divided into five parts: the follicle stimulation protocol, pituitary suppression, luteal phase support, pretreatment regimens, and supplementary support.

In our study, we compared two protocols. Neither of these protocols included supplementary support, and both had the same luteal phase. We used the same gonadotropins and GnRH agonists. Our standard protocol was based on OC pretreatment from the 2nd to 4th day, which is considered a long protocol with mild pituitary suppression, using 0.1 mg triptorelin every 2 days. The stimulation was based on urinary gonadotropins (Menopur, Ferring, Saint-Prex, Switzerland). For this investigation, we decided to use 150–225 IU for each patient. The comparison protocol was based on different pretreatments and stimulations. We used estradiol after ovulation during the previous natural cycle and started controlled ovarian stimulation, using a short protocol with an agonist for pituitary suppression. We chose this protocol because of the increasing number of studies showing that almost the same results can be achieved using short antagonist protocols compared with long agonist protocols. Short protocols are strongly favoured for several reasons, some of which are medical and some of which are social, such as requiring a fewer number of injections, which makes these IVF programmes more patient friendly. It was found that the pituitary suppression resulting from the use of combined OC in GnRH antagonist cycles is associated with slower follicular growth and lower serum estradiol levels during the early part of the cycle [2].

Most of the studies reports have been based on antagonist protocols. In the Cochrane Review, Al-Inany considered forty-one trials reporting clinical PRs in 6571 women. There was a significant difference in the clinical PRs following GnRH antagonist protocols compared with GnRH agonist protocols (OR = 0.84, 95% CI = 0.75 to 0.94; *P* = 0.002; I2 = 0.00%, 95% CI = 0.00% to 0.00%) [5]. Most major reviewed studies, such as Euro Orgalutran [6] (486 cases) and Sbracia et al. [7] (283 cases), have reported significantly higher PRs with long agonist protocols compared with antagonist protocols (OR = 0.70, 95% CI = 0.49 to 1.00 and OR = 0.47, 95% CI = 0.28 to 0.79, resp.). We did not find any published studies that compared a long agonist protocol with estradiol valerate pretreatment short agonist protocol.

Our main outcome of interest, the PR, was significantly different by more than 18%. This rate is consistent with our preliminary study. Other results may partly explain this phenomenon.

The significantly shorter duration of stimulation and use of hMG in short protocols are most likely influencing factors. Furthermore, the weak pituitary gland suppression by the agonists in both protocols (combined OC pills and estradiol) and the use of triptorelin at the beginning of stimulation allow for a more rapid follicle reaction, which decreases the total gonadotropin consumption.

A surprising finding from this study was that we obtained a smaller number of oocytes with the short protocol than with the long protocol. This result, however, was also mentioned in a 2014 article by Sunkara et al. [8]. This finding may be potentially explained by a lack of ovarian suppression that occurs using pretreatment with orally administered 4 mg estradiol, which may partially cause the selection of dominant follicles. Selection refers to the process by which the maturing follicular cohort is reduced to a number that is appropriate for the species-specific ovulatory quota. This process involves a negative selection against the subordinate follicles and a positive selection of the follicles that will determine dominance [9]. The nonuniform growth of the follicles in our short protocol may have caused a much more diverse maturation of the retrieved oocytes and may explain the 10% lower fertilisation rate of the oocytes from the estradiol short protocol cycles. As a consequence, we obtained significantly fewer blastocysts for transfer. Adjusting the long protocol results for a comparable number of transferred blastocysts may partially explain the PR difference. This adjustment cannot, however, explain the difference in the implantation rate between the groups. This difference may have been caused by the quality of the transferred embryos, which may not be optimal in cycles that are not suppressed by estradiol agonists. We excluded the detrimental effects of agonists on the endometrium because the same triptorelin was used in the long protocol cycles. We also excluded the deleterious effects of estradiol on the endometrium. Some previous studies have compared estradiol pretreatment with no treatment in antagonist cycles [10, 11].

We still believe that there are opportunities for agonist short protocol implementation. We propose two different future studies. One study is a comparison of short and long protocols after combined OC pill pretreatment. This future study may elucidate which of the effects observed in the current study depended on the different pretreatments and which effects depended on the longer influence of the GnRH agonist on the endometrium and ovaries. The disadvantages of this short protocol include the same long period between the start of treatment and the embryo transfer and the need to take OC. The other future study, which seems more interesting, will aim to determine the optimal estradiol dosage that will decrease FSH and LH levels and suppress follicle selection and dominance. According to Cedrin-Durnerin, estrogen administration (at a dosage of 4 mg/day) did not suppress serum LH and FSH levels [2], but another study indicated that estrogen administration should decrease FSH levels [12]. A potential solution for suppression of gonadotropins is the vaginal administration of estradiol, which results in a serum estradiol level that is a few times higher than the level achieved with oral administration (unpublished data). This finding would be inconsistent with the recent data published by Chen et al. [13], which suggested that a high concentration of estradiol is detrimental to endometrial glandular cells. Our previous results are inconsistent with those recent findings showing increasing pregnancy rate during estradiol supplementation of luteal phase [14].

## Figures and Tables

**Table 1:** Characteristics of the treatment groups.

Variable	Long agonist protocol with OC pretreatment	Short agonist protocol with vaginal estradiol pretreatment	*P* value
Number of subjects	154	144	—
Number of cycles	157	158	—
Mean (SD) age	32.5 (3.97)	32.5 (2.96)	0.68
95% CI	(31.9–33.1)	(32.1–33.1)	
BMI (kg/m^2^) (SD)	22.2 (1.3)	22.4 (1.1)	0.16
Tubal factor (%)	44 (27.5)	47 (29.9)	—
Male factor (%)	58 (36.2)	55 (35.0)	—
Anovulation (%)	28 (17.5)	23 (14.6)	—
Unexplained (%)	30 (18.8)	32 (20.4)	—
Mean (SD) duration of infertility (years)	4.2 (2.8)	4.3 (2.9)	0.82
AMH (SD) (ng/mL)	3.2 (2.5)	3.3 (2.9)	0.97
95% CI	(2.8–3.5)	(2.9–3.8)	
Inhibin B (SD)	60.9 (42.3)	67.2 (37.4)	0.14
95% CI	(52.3–69.5)	(60.8–73.4)	
Basal FSH (SD)	7.4 (2.3)	8.1 (2.1)	0.32
Basal LH (SD)	7.2 (1.8)	7.1 (1.6)	0.23
Basal E2 (SD)	47.1 (13.2)	47.5 (11.1)	0.71
DHEA-S (SD)	204.6 (20.3)	207.5 (17.8)	0.57
Testosterone (SD)	1.3 (0.6)	1.6 (0.4)	0.07
SHBG (SD)	81.3 (18.4)	67.9 (21.3)	0.11
AFC (SD)	17.4 (10.1)	18.8 (12.5)	0.58
95% CI	(15.9–19.1)	(16.7–20.8)	

Values are *n* (%) or means ± SDs. AMH: anti-Müllerian hormone; BMI: body mass index; AFC: antral follicle count; DHEA-S: dehydroepiandrosterone sulphate; SHBG: steroid hormone-binding globulin.

**Table 2:** In vitro fertilisation programme characteristics of the investigated groups.

Variable	Long agonist protocol with OC pretreatment	Short agonist protocol with vaginal estradiol pretreatment	*P* value
Number of cycles	157	158	—
Number of transfers	137	134	—
Duration of stimulation, days (SD)	9.8	8.1	0.03
hMG dose (IU)	1861.5	1755	0.04
Number of oocytes retrieved (SD)	7.8 (4.3)	6.9 (4.3)	0.05
Fertilisation rate (%)	68.5	57.9	0.003
Number of embryos transferred	1.8	1.4	<0.001
Pregnancy rate (per ET, %)	80 (58.4)	54 (40.3)	0.003
Implantation rate (%)	37.8	28	0.03
Multiple pregnancy rate (%)	23 (35.4)	9 (21.4)	0.12
Ectopic pregnancy (%)	0	0	—
OHSS (%)	0	0	—
Spontaneous abortion rate (%)	12 (15)	11 (20.4)	0.81
